# Supplementary material for: Chromothripsis during telomere crisis is independent of NHEJ, and consistent with a replicative origin
Source: Genome Res. 2019 May;29(5):737–49. doi: 10.1101/gr.240705.118 (PMC6499312; doi:10.1101/gr.240705.118)
Supplement: Supplemental Material [file supp_gr.240705.118_Supplemental_file_1.zip › contigs/annotated_contigs/DB41/contig.3.DB41_length_557_mean_cov_14.5619389587.docx]

**DB41_length_557_mean_cov_14.5619389587**

```
TTTTCAACCCAGAATATCATATCCTGCCAAACTAGCTTCATAAGGGAAGGAGTAATAAAATCCTTTACAGACAAGAAAATGCGGAGAGA
 >chr5:119325811-119326139 - E=1e-167
TTTTGTCACCACCAGGCCTGCCTTACAAGGTCTCCTGAAGGACGTACTAAACATGGAAAGGAACAACTGGTACCAGCCACTGCAAAAAC

ATGCCAAATTGTAAAGACCATCAATGCTATGAAGAAACTGCATCAATTAACAGGTGAAATAACCAGCTAGCATCATAATAACAAGATCA

AATTCACACATAACAATATTAACCTTAAATGTAAACGGGCTAAATGCCCCAATTAAAAGAC|TTAGACTGGCAAACTGGATCTTTATCA

AC|TCAAGACCCATTAGTGTGCTGTATTCAGGAGACCCATCTCATGTGCAAAGACACACATAGGCTCAAAATAAAGGGATGGAGGAAGA
   >chr5:120984206-120984352 - E=4e-61 p=0e+00
TTTACCAAGCAAATGGAAAGCAAAAAAAAAAAAAAAAAAAAAAAAAAAAAAAAAAAAAAA|GGGAAGCAATAATAAAATAAG|AAAAAA
                                                                                  >chr3
AAAACACCAAAATAAAAAAAAAAAAA|A:101310588-101310620 - E=1e
```
